# Risk management in the dual crisis of unresectable colorectal cancer and deep vein thrombosis: navigating between hemorrhage and antithrombosis

**DOI:** 10.3389/fonc.2026.1758399

**Published:** 2026-03-20

**Authors:** Xiaoqing Wu, Hongxuan Tong, Jing Li

**Affiliations:** 1 Oncology Department, Wangjing Hospital of China Academy of Chinese Medical Sciences, Beijing, China; 2 Institute of Basic Theory for Chinese Medicine, China Academy of Chinese Medical Sciences, Beijing, China

**Keywords:** anticoagulation strategy, clinical decision-making, colorectal cancer, deep vein thrombosis, gastrointestinal bleeding

## Abstract

Patients with advanced, unresectable colorectal cancer in need of anticoagulation for deep vein thrombosis (DVT) with active or high-risk gastrointestinal bleeding (GIB) have a critical dilemma. Withholding anticoagulation has a risk of fatal thrombosis, while giving anticoagulation makes life-threatening bleeding worse. A retrievable inferior vena cava (IVC) filter may be a temporary stopgap while an active GIB is occurring. Once hemostasis has occurred, therapeutic anticoagulation should begin. Low molecular weight heparin (LMWH) is preferred over direct oral anticoagulants (DOAs) because of a lower risk of mucosal bleeding, and if necessary, apixaban is suggested. Dosing must be adjusted for thrombocytopenia. Management requires meticulous and individualized risk assessment. Future priorities include improved risk-prediction tools and targeted therapies to mitigate this precarious “therapeutic tightrope.”

## 1 Introduction: walking the therapeutic tightrope

Colorectal cancer (CRC) is the third most common malignancy and the second most common cause of death due to cancer worldwide, with 1.93 million new cases and about 904,000 deaths reported in 2022 (1). The type of cancer has a significant impact on the risk of VTE, with the greatest risk being seen in pancreatic cancer, gastrointestinal cancer, and brain cancer, and the lowest risk in breast cancer, prostate cancer, endocrine cancer, and skin cancer (2). It is interesting to note that CRC shows this connection, as there have been reports that indicate CRC has one of the highest incidences of thrombotic events compared to other common cancers (3). Overall, 4 to 7 times higher risk for VTE, three times higher risk of recurrent VTE, two times higher risk of bleeding due to anticoagulation, and ten times higher risk of death are compared to patients without cancer (4). Patients often experience the weakening effects of the disease progression, including malnutrition (5), cancer-related fatigue that leads to mobility problems (6), and hypercoagulability, among other direct and indirect effects of the tumor. Cancer-associated thrombosis (CAT) is a common complication in patients with cancer, with it being the second leading cause of death (7). As many as 20% of patients with VTE are diagnosed at the same time with cancer, and patients who have cancer have a much higher risk of VTE, 14-25% (4, 8, 9). In addition, CAT is a significant contributor to morbidity and mortality, where VTE is the second leading cause of death in cancer patients (after cancer) (10, 11). In this vulnerable population, there are two specific complications: gastrointestinal bleeding (GIB) and VTE, which often coincide. This is a very significant and dangerous therapeutic challenge for clinicians: DVT is the most common. This co-morbidity is one of the most dangerous “tightrope” situations in the treatment of cancer.

The pathophysiological features of unresectable CRC make the patients susceptible to both risks. Tumors, especially in the gastrointestinal mucosa, are delicate and prone to bleeding, which is worsened by the usual treatment, such as chemotherapy and antiangiogenetic agents (e.g., bevacizumab). Simultaneously, cancer-associated hypercoagulability (Trousseau syndrome) is a major instigator of VTE caused by hypercoagulability via tumor procoagulant expression, endothelial activation, cytokines, and patient inactivity associated with inflammation (12). Consequently, the prevalence of GIB and VTE co-morbidity is significantly higher in this group than in the general population or other patients with cancer.

When these two life-threatening conditions happen simultaneously or occur close to each other in time, a conundrum of clinical decision-making arises. For newly diagnosed DVT, the standard of care includes therapeutic anticoagulation, i.e., with heparin analogs (low molecular weight heparin (LMWH) is a key component, and increasingly the direct oral anticoagulants (DOACs) are being used instead) to prevent fatal pulmonary embolisms (PEs), stroke, or clot progression (13). However, using these powerful anticoagulants in patients who are bleeding or at high risk for recurrent bleeding due to tumor activity, frailty, or recent hemorrhage significantly raises the risk of uncontrolled GIB, potentially leading to catastrophic bleeding, shock, emergency intervention, and death.

On the other hand, if anticoagulation is declined or disrupted in cases of severe DVT, patients face a risk of thromboembolic complications. It is widely recognized that tumor-associated thrombi tend to develop and recur; without treatment, the risk of a potentially fatal thromboembolic or debilitating ischemic event increases significantly. This risk is heightened in the physiologically fragile condition of advanced malignancy (1).

## 2 Core dilemma analysis: anticoagulation and hemorrhage - a precarious balance

Patients with CRC experience severe cancer-related hypercoagulation. The expression of tumor tissue factor (2, 3), release of extracellular vesicles (4), treatment-induced endothelial damage (e.g., chemotherapy, antiangiogenic drugs like bevacizumab) (5), along with common sequelae (e.g., mobility issues and inflammation), leads to a significantly higher incidence of VTE compared to the general population and patients with many other solid tumors (6). DVT is not just a complication, but a large source of death in non-cancer patients as well. It carries a significant risk of PE, post-thrombotic syndrome, and requires a disruption of treatment.

**Figure 1:**
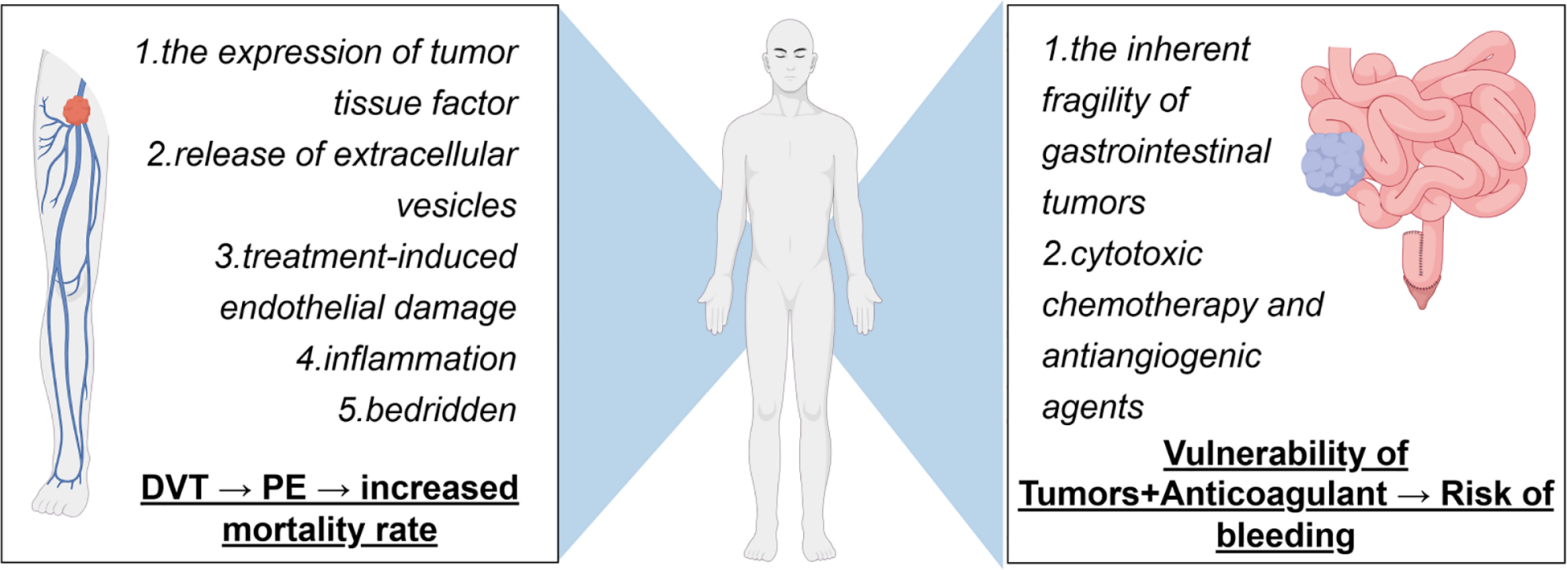
The anticoagulation-bleeding balance dilemma in patients with unresectable colorectal cancer and deep vein thrombosis.

Moreover, CRC destroys the integrity of the gastrointestinal tract. Luminal tumor is fragile and prone to erosion. Progressive disease, malnutrition, cytotoxic therapies, and the antiangiogenic effects of targeted medications may compromise the mucosal barrier, which leads to persistent bleeding. A proportion of patients will have clinically significant GIB, which ranges from occult anaemia to a life-threatening haemorrhage requiring urgent endoscopic, interventional, or surgical intervention. Common causes of DVT and bleeding are shown in **Figure 1**.

Evidence-based guidelines provide clear but isolated recommendations for CAT. Aggressive therapeutic-dose anticoagulation is necessary for CAT. Major society guidelines [ASCO (7), ISTH (8, 9), NCCN (10), ACCP (11)] recommend LMWH as the initial gold standard therapy, with DOACs as an alternative, while noting the GIB risk in intraluminal GI cancers. LMWH (e.g., enoxaparin, nadroparin calcium, and dalteparin sodium) continues to be the preferred option for gastrointestinal tumors with unresected primary lesions. The CLOT study demonstrated that LMWH decreases the risk of VTE recurrence by 50% compared to warfarin (9% vs. 17%) (12). Owing to the absence of metabolic conversion and direct gastrointestinal irritation, their risk of mucosal bleeding is much lower than that of DOACs. Nevertheless, the need for long-term injections leads to a patient compliance rate of just 37%, compared to 61% for those on oral medications, with dose adjustments required for patients with renal impairment (13). Active GI hemorrhage completely rules out the use of therapeutic anticoagulants. Standard protocols highlight the need for swift hemostasis, resuscitation, and the suspension of anticoagulants until bleeding is managed and the risk of rebleeding is substantially minimized.

Here lies the unsustainable paradox. Patients with CRC often face both needs: Clinically significant DVT necessitates anticoagulation to prevent potentially fatal thromboembolism. The risk of GIB that is ongoing or significant, particularly because of recent active bleeding, visible fragile tumors, high-risk anticancer therapy, or underlying mucosal weakness, can make anticoagulation uncertain.

### 2.1 Peril of therapeutic anticoagulation

Therapeutic anticoagulation using LMWH or DOACs forms the foundation of CAT management as per international guidelines (7–10). However, the mechanisms of these agents, involving inhibition of Factor Xa or thrombin, may impair hemostasis at sites of vascular fragility. DOACs may also induce GI bleeding by a local anticoagulant effect exercised on the gastrointestinal mucosa due to a portion of drug that is unabsorbed and that remains inside the intestinal lumen (14–16). Based on current evidence, LMWH may be a preferable option for cancer patients at high risk of gastrointestinal bleeding, including those with active gastrointestinal tumors (17). The latest guidelines indicate that the bleeding risk associated with DOACs is comparable to or higher than that of dalteparin, depending on the specific drug type and tumor location. While there is no significant difference between the two drugs in terms of major bleeding risk, DOACs carry a higher risk of clinically relevant bleeding, encompassing both major and non-major bleeding events. In CRC, fragile tumor vasculature, mucosal ulcerations caused by chemotherapy or anti-angiogenic agents (e.g., bevacizumab) (25), and pre-existing bleeding lesions form high-risk substrates. Antiplatelet therapy is not recommended. Results from a retrospective study indicate that independent predictors of major bleeding include age ≥75 years, history of major bleeding, ECOG performance status ≥2, antiplatelet therapy, and platelet count <100 × 10^9^/L (18).

Strong evidence shows that major bleeding rates are higher in gastrointestinal (GI) cancers during anticoagulation. The HOKUSAI-VTE Cancer trial demonstrated a significantly higher incidence of major GIB with edoxaban compared to dalteparin in GI cancer subgroups (6.5% vs. 3.6%; HR 1.62, 95% CI 1.00–2.63) (19). Data further show that major GIB occurs in 46% of anticoagulated GI cancer patients within 6 months (20). A key mechanistic explanation for the higher GIB risk with DOACs, as suggested by trials like HOKUSAI-VTE Cancer, involves local drug exposure. A portion of unabsorbed DOAC excreted into the GI lumen may exert a topical anticoagulant effect on the fragile, often ulcerated mucosa of GI tumors, a mechanism not shared by parenteral LMWH. However, studies have also shown that patients using DOACs for anticoagulation have a lower bleeding rate than those using LMWH. The proportion of patients requiring hospitalization for bleeding within 12 months of initiating DOAC therapy was 6.6%, compared to 8% for LMWH and 8.6% for warfarin.

(21). It must be noted that this data does not pertain to the gastrointestinal tumor subgroup; the included population encompasses multiple tumor types including central nervous system, gastrointestinal, prostate, kidney/bladder, lung, lymphoma, and melanoma. Some physicians also highlight the advantages of LMWH over DOACs in high-risk GIB patients from another perspective: LMWH has a shorter half-life (approximately 12 hours) and specific antagonists available for severe bleeding episodes (e.g., protamine sulfate can partially reverse its effects). Although DOACs now have corresponding reversal agents, LMWH has a longer history of clinical use, making physicians potentially more familiar with its management in emergency situations.

Importantly, bleeding risk is not uniform across GI cancers but is further modulated by the specific anatomic location of the tumor.​​ The highest GIB rate in colorectal cancer patients belonged to hepatic flexure tumors (11.1%), and the lowest GIB was for descending colon cancer (8.9%). Patients with GIH were less likely to be females (37.8% *vs* 43.3%, *P* < 0.001). While minority races, including Black, Hispanic, Asian, and Native American, were more prevalent in patients with GIB, White race was less common in GIB patients (63.0% *vs* 68.3%, *P* < 0.001) (23). Additional studies indicate that among patients with multiple tumor types receiving anticoagulant therapy, the cumulative bleeding incidence is highest in those with unresected lower gastrointestinal tract cancers (21). In the CARRAVAGIO trial, 36.5% of major bleeding events occurred in patients with gastrointestinal tumors (upper gastrointestinal tract: 11.1%; lower gastrointestinal tract: 24.4%), and all bleeding events in gastrointestinal tumor patients occurred in those with unresected tumors (n = 16/16) (24). Consequently, initiating full-dose anticoagulation risks hemorrhagic transformation of subclinical lesions, refractory bleeding requiring endoscopic or surgical intervention, hemodynamic instability, transfusion dependency, and hemorrhagic shock.

Consequently, initiating full-dose anticoagulation, while guideline-directed, risks hemorrhagic transformation of subclinical lesions, leading to refractory bleeding, invasive interventions, and hemodynamic compromise. This peril underscores the limitation of current first-line agents and highlights a critical therapeutic gap for patients with high bleeding risk tumors.

### 2.2 Peril of anticoagulation avoidance

Cancer patients exhibit a high thrombogenic potential owing to various pathological mechanisms. Malignant cells release procoagulant factors such as tissue factor and cancer procoagulant (3). Cytotoxic therapies exacerbate vascular endothelial injury (25). Decreased mobility and systemic inflammatory reactions also play a role (26). Without treatment, proximal DVT advances to PE in more than 40% of cases within weeks (27, 28). Compared to shorter therapy durations (3–6 months), longer anticoagulation therapy (>6 months) likely decreased the incidence of nonfatal PE and DVT. Long-term anticoagulation and antiplatelet therapy are linked to a reduction in all-cause mortality compared to discontinuing treatment in patients with a history of DVT (29). This risk profile is especially evident in CRC, where K-ras mutations increase plasminogen activator inhibitor-1, further enhancing thrombogenesis (30).

The clinical outcomes of suboptimal anticoagulation can be serious. Acute mortality reaches approximately 30% in cancer patients with massive PE (31). Cerebral infarction can occur as a result of paradoxical embolization. Persistent post-thrombotic complications arise in 40% of treated cases (32). These data highlight the pressing need for timely and appropriate thromboprophylaxis in high-risk groups.

### 2.3 Inadequacy of compromise strategies

Common “middle-ground” approaches show limited efficacy: As shown in **Table 1**.

**Table 1:** Current clinical management strategies for patients with unresectable colorectal cancer and deep vein thrombosis.

Strategy	Key limitations	Evidence
​Sub-therapeutic anticoagulation​	Fails to prevent thrombus propagation	a. Treating patients with a longer course (>6 months) of anticoagulation vs a shorter course (3–6 months), reduced recurrent PE (risk ratio [RR], 0.66; 95% confidence interval [CI], 0.42-1.02) and DVT (RR, 0.85; 95% CI, 0.63-1.14) (29).
​Inferior vena cava Filters​ (IVCF)	IVC thrombosis No effect on DVT progression •Requires eventual AC	a. An overall recurrent DVT rate of 5.4% in patients with IVC filters; The incidence of IVC thrombosis or stenosis was 2.5% (33). b. Patients should be accompanied by subsequent anticoagulation once the patient can safely be given anticoagulant drugs (34).

## 3 Management strategies: from genetic risks to clinical decision optimization

Several factors have an impact on anticoagulation results in cancer patients. BAIAP2L2, CYP3A5, ABCG2, and VWF could be associated with bleeding in patients treated (43). The clinical state of patients plays a very significant role in therapeutic decisions. Patients with poor functional status or limited predicted survival are seen to have a lower clinical benefit-to-bleeding risk ratio. Treatment goals are also a guide to management approaches. For patients who cannot undergo curative surgery due to physical condition, tumor stage, or other factors and can only receive palliative care, balancing benefits and risks becomes particularly crucial. These variables call for individualized anticoagulation protocols in the oncologic practice.

When there is an interaction between the risk of severe thromboembolism and the risk of bleeding, such as in the case of free inferior vena cava thrombosis (Forrest Ia type) with active bleeding ulcers, a retrievable inferior vena cava filter may be implanted first. Once hemostasis is secured and hemodynamic stability is confirmed, treatment can be converted to full-dose LMWH therapy. The choice of anticoagulant drugs may also impact the risk of bleeding during the healing process of the lesion. In situations with low thrombus burden but high risk of bleeding, as in cases of deep vein thrombosis in a distal location with tumors in the gastrointestinal tract without primary lesion resection, the adjunct to mechanical prophylaxis of the patient is ultrasound monitoring every 2 weeks, and drug therapy is reserved for situations when thrombus progression exceeds 3 cm. This layered approach guarantees specific management based on thrombus urgency and bleeding susceptibility. In cases where patients exhibit thrombocytopenia (observed in 47% of hematologic malignancies and 22% of solid tumors), the intensity of anticoagulation should be modified based on the severity grade.

For patients with active GIB complicated by DVT, clinical management requires careful balancing of hemostasis and anticoagulation. Treatment decisions should be stratified based on the patient’s risk of pulmonary embolism and the severity of bleeding. The following are recommended strategies for this clinical scenario (7, 10, 28): As shown in **Table 2**.

**Table 2:** Hierarchical framework for anticoagulation decision-making in acute GIB with DVT.

Clinical senario	Risk of PE	Degree of GIB	Clinical strategy
Patients with actively GIB combined with DVT	Any risk	Fatal or uncontrollable bleeding	a. Withhold anticoagulation. b. Actively manage the source of bleeding, administer medication for hemostasis, and perform endoscopic hemostasis or selective embolization of arteries when necessary.
	High-Risk (e.g., proximal DVT)	Major/Active, but Controllable Bleeding	a. Withhold anticoagulation; prioritize bleeding control. b. Strongly consider a temporary (retrievable) IVCF​ to prevent PE during the high-risk period. c. Close monitoring​ for thrombus extension (ultrasound) and signs of PE. d. Restart therapeutic-dose anticoagulation​ as soon as hemostasis is secure.
	Low-Moderate Risk​ (e.g., isolated distal DVT)		a. Withhold anticoagulation; prioritize bleeding control.​​ b. IVCF may be deferred​ due to lower thrombotic risk. c. Close clinical and ultrasound monitoring. d. After hemostasis, consider restarting with a ​prophylactic or intermediate dose (it is recommended to begin by administering unfractionated heparin intravenously.), then escalate to therapeutic dose.
	Any Risk	Minor/Clinically Insignificant Bleeding	a. Treat bleeding locally in combination with PPI therapy. b. Consider temporarily discontinuing medication or reducing anticoagulant intensity. c. Resume therapeutic-dose anticoagulation as soon as possible.

For patients with unresectable colorectal cancer complicated by deep vein thrombosis (DVT), the decision-making and management of palliative surgery constitute a multi-stage process centered on balancing the risks of thromboembolism, tumor-related complications, and surgical bleeding. This framework integrates and proposes feasible management pathways for this high-risk population (11, 37–40): As shown in **Table 3**.

**Table 3:** Timing of palliative surgery and perioperative anticoagulation management in patients with unrescetable colorectal cancer and DVT.

Clinical scenario	Clinical strategy
Timing of surgery	a. For patients eligible for palliative surgery, administer therapeutic-dose anticoagulation for at least 5–7 days prior to surgery. b. Preoperative placement of a retrievable IVCF
Peri-operative management considerations	a. Discontinue the last therapeutic dose of LMWH 24 hours prior to surgery (40, 41). b. Initially resume LMWH at a prophylactic dose. LMWH bridging therapy should be resumed 48 to 72 hours postoperatively, with the initial 2 to 3 days administered at the lowest dose (40).

The combination of DOACs and proton pump inhibitors (PPIs) (e.g., omeprazole and esomeprazole) can reduce the risk of gastric ulcers, and thus the risk of bleeding events is decreased by 37%. A randomized placebo-controlled trial found that the use of proton pump inhibitors in patients receiving low-dose anticoagulation and/or aspirin may reduce bleeding from gastroduodenal lesions (22). Mechanistically, PPIs inhibit gastric acid secretion, increase platelet aggregation, and promote mucosal repair. The SELECT-D study demonstrated that the clinically relevant bleeding rate of rivaroxaban in gastrointestinal tumors was 13% (4% in the LMWH group), especially in upper gastrointestinal tumors (35). Apixaban or LMWH is suggested as an alternative option. The CARAVAGGIO study found a major bleeding rate of 3.5%, which is lower than the risk associated with rivaroxaban (36).

DOACs (such as rivaroxaban and apixaban) are substrates of CYP3A4 and P-gp. Clarithromycin, erythromycin, and fluconazole significantly increase the blood concentration of DOACs by inhibiting these two pathways, leading to an increased risk of bleeding. The risk of gastrointestinal bleeding within 7 days after DOAC combined with erythromycin increased by 3.66 times (99% CI: 1.27–10.51). The case-crossover analysis of rivaroxaban combined with erythromycin showed a 38% increase in the risk of clinically relevant non-major bleeding. The combined use of DOACs and fluconazole was associated with a 2.90-fold increase in all-cause mortality risk (99% CI: 1.74–4.83) (42, 43).

## 4 Future directions and imperatives

Despite gradual advancements, the risk-benefit balance between thrombosis and bleeding in patients with unresectable CRC still demands transformative solutions. Future risk assessment must evolve beyond static clinical scores by dynamically integrating multimodal data. Promising biomarkers include ​D-dimer kinetics​ for thrombotic propensity, ​circulating VEGF​ for vascular fragility, and ​inflammatory markers​​ for systemic driver of complications. The true potential lies in leveraging ​AI and machine learning​ to synthesize these dynamic biomarkers with real-world clinical variables (tumor burden, prior bleeding, renal function). Such models can generate continuously updated, individualized risk forecasts, identifying not only who is at high risk, but when that risk is highest, enabling pre-emptive intervention. Secondly, conducting definitive trials in this high-risk, dual-pathology population requires innovative designs that are both ethical and efficient. ​AI-enhanced adaptive platform trials​ represent a promising avenue. Such trials can seamlessly compare multiple strategies (e.g., full-dose vs. reduced-dose anticoagulation, with or without IVC filter bridging). AI algorithms can analyze incoming outcome data to dynamically adjust randomization probabilities, preferentially assigning patients to the currently most promising therapeutic arm, thereby accelerating learning and minimizing exposure to less effective options. Third, Fourth, AI-driven pharmacokinetic/pharmacodynamic modeling, informed by continuous biomarkers like ​anti-Xa activity​ or ​thrombin generation profiles, could guide real-time, personalized anticoagulant dosing to maintain efficacy while avoiding dangerous troughs or peaks. On a translational level, AI can accelerate the discovery of novel drug targets by analyzing omics data to identify key drivers of cancer-associated coagulopathy. This paves the way for next-generation agents, such as engineered nanoparticles designed to selectively inhibit tumor-derived procoagulant factors, potentially uncoupling antithrombotic protection from systemic bleeding risk. Emerging evidence supports the value of such models: A machine learning model for assessing VTE risk after CRC surgery was developed and trained using stacking ensemble technology with six machine learning algorithms. All models were developed through 10-fold cross-validation on the training set. APTT, Caprini score, D-dimer, Hb, PA, surgical site, and surgical type were all statistically significant in multivariate analysis (P < 0.05) (44). A retrospective study analyzed the risk of deep vein thrombosis in patients after colorectal cancer surgery. It used a combination of random forest and XG boost algorithms, LASSO regression with univariate regression analysis to find the significant predictors, which were age, preoperative albumin, preoperative hemoglobin, operative time, PIKVA2, CEA, and preoperative neutrophil count. Age and preoperative albumin were the main factors that determined ML model predictions (49). Further multisource collaboration on data is required to refine feature selections and ensure generalization.

The importance of shared decision-making between the patient and the physician should be emphasized: in such “lose-lose risk” dilemmas, clear and honest communication with patients and families is important. Discuss the prognosis of the disease, important risks of each option, and individual aspects of the final choice.

## 5 Conclusion

The simultaneous management of VTE and GIB in patients with CRC is one of the most dangerous clinical problems in oncology. This comment emphasizes the profound and often unsustainable paradox faced by these patients: while therapeutic anticoagulation is essential to prevent a fatal thromboembolic event, it raises the risk of hemorrhage due to the fragility of the neovascularization or mucosal injury and, on the other hand, anticoagulants if they are not anti-coagulated, they are at risk of a catastrophic progression of the thrombosis as well as pulmonary embolism and death.

Current strategies, which include sub-therapeutic anticoagulation or intravenous venous collection devices (IVC filter), offer limited protection from thrombosis and expose patients to further risks. Personalized risk stratification based on thrombus burden (proximal vs. distal DVT), bleeding lesion severity (e.g., according to Forrest classification), profiles of biomarkers (e.g., TFPI, fibrin monomer), and platelet counts are critical. There is evidence for a tiered approach: retrievable IVC filters in those with high thrombus risk in the presence of active bleeding (transition from IVC to LMWH after hemostasis), LMWH (preferred over DOACs) in patients with unresected GI tumors to prevent mucosal bleeding, and apixaban plus lesion to proton-pump inhibitor in select cases. Drug interactions, e.g., between DOACs and CYP3A4/P-gp inhibitors, need to be carefully managed. Until transformative solutions are seen, clinicians will need to perform this therapeutic tightrope act with prudent judgment - to find a balance between life-threatening situations in the present with long-term survival, and to continue to pursue personalized, evidence-based equipoise.

## Data Availability

The original contributions presented in the study are included in the article/supplementary material. Further inquiries can be directed to the corresponding authors.
